# Modification of a Method for Diagnosing Noise-Induced Hearing Loss Sustained During Military Service

**DOI:** 10.1177/23312165221145005

**Published:** 2022-12-14

**Authors:** Brian C.J. Moore, Larry E. Humes, Graham Cox, David Lowe, Hedwig E. Gockel

**Affiliations:** 1 Cambridge Hearing Group, Department of Psychology, 2152 University of Cambridge, Cambridge, UK; 2 Department of Speech, Language and Hearing Sciences, Indiana University, Bloomington, IN, USA; 3 ENT Department (retired), 6397 Oxford University Hospitals NHS Foundation Trust, Oxford, UK; 4 ENT Department, 156705 James Cook University Hospital, Middlesbrough, Cleveland, UK; 5 Cambridge Hearing Group, MRC Cognition and Brain Sciences Unit, 2152 University of Cambridge, Cambridge, UK

**Keywords:** noise exposure, hearing loss, military service, diagnosis, sensitivity and specificity

## Abstract

Moore (2020) proposed a method for diagnosing noise-induced hearing loss (NIHL) sustained during military service, based on an analysis of the shapes of the audiograms of military personnel. The method, denoted M-NIHL, was estimated to have high sensitivity but low-to-moderate specificity. Here, a revised version of the method, denoted rM-NIHL, was developed that gave a better balance between sensitivity and specificity. A database of 285 audiograms of military noise-exposed men was created by merging two previously used databases with a new database, randomly shuffling, and then splitting into two, one for development of the revised method and one for evaluation. Two comparable databases of audiograms of 185 non-exposed men were also created, again one for development and one for evaluation. Based on the evaluation databases, the rM-NIHL method has slightly lower sensitivity than the M-NIHL method, but the specificity is markedly higher. The two methods have similar overall diagnostic performance. If an individual is classified as having NIHL based on a positive diagnosis for either ear, the rM-NIHL method has a sensitivity of 0.98 and a specificity of 0.63. Based on a positive diagnosis for both ears, the rM-NIHL method has a sensitivity of 0.76 and a specificity of 0.95.

## Introduction

Noise-induced hearing loss (NIHL) is commonly diagnosed based on the presence of a notch or bulge in the audiogram centered near 4 kHz (Coles et al., 2000; Niskar et al., 2001; Phillips et al., 2010; Pudrith et al., 2022). Moore (2020) argued that such methods are not appropriate in cases of NIHL sustained during military service, which often involves exposure to very intense impulsive sound and does not always produce a clear notch or bulge in the audiogram. Moore proposed a diagnostic method called M-NIHL. Based on analyses of the audiograms of former military personnel claiming compensation for NIHL sustained during military service, the M-NIHL method was shown to have high sensitivity, defined as the proportion of cases with NIHL that are correctly diagnosed (Lowe & Moore, 2021; Moore, 2020). However, the specificity, defined as the proportion of cases without NIHL that are correctly diagnosed, estimated using a matched population that was carefully screened to exclude individuals with possible noise exposure, was only moderate (Moore & von Gablenz, 2021). This paper proposes a modified version of the M-NIHL method, denoted rM-NIHL. The modified method has slightly lower sensitivity than the M-NIHL method, but the specificity is markedly higher, giving a better balance between sensitivity and specificity.

The “ideal” balance between sensitivity and specificity depends on the purpose of making a diagnosis of NIHL. If the diagnosis is made to support a claim for compensation for NIHL, then to be fair to the individual it is important that a reasonably high proportion of individuals who do have NIHL receive a positive diagnosis. This must be balanced against the need to avoid payment of compensation to individuals who do not have NIHL. In a medico-legal context, the criterion is usually the “balance of probabilities,” i.e., for a positive diagnosis to be made, it should be more likely than not that the individual has NIHL. However, this depends not just on the audiogram, but on other factors, as discussed below. Another complication is that exposure to noise during military service often results in greater hearing loss in one ear (usually the left) than the other (Keim, 1969; Lowe & Moore, 2021; Moore, 2020). Often, the asymmetry can be related to asymmetry of the exposure (Keim, 1969; Lowe & Moore, 2021). Hence, it is possible for a diagnosis of NIHL to be positive for one ear but not for the other ear. In such cases, the question arises as to whether diagnosis of an *individual* as having NIHL should depend on a positive diagnosis for both ears or on a positive diagnosis for either ear or both. In the opinion of the authors, the latter is more appropriate. However, in the present paper, both cases are considered. Compared to the case of a positive diagnosis for both ears, an either-ear criterion for positive diagnosis is expected to have greater sensitivity but lower specificity. Our goal was to develop a method with an appropriate balance of probabilities, defined as specificity greater than 0.6 based on a positive diagnosis for either ear with sensitivity greater than 0.75 based on a positive diagnosis for both ears.

To make a diagnosis of NIHL incurred during military service, it is necessary to assess whether there is any other plausible cause of hearing loss, including noise exposure outside military service. This requires a thorough medical history to be obtained for the individual concerned (Moore et al., 2022). In what follows, it is assumed that such a history has been obtained and that causes of hearing loss other than noise exposure have been deemed to be unlikely.

The M-NIHL method has four requirements, denoted by R and a number, and in the case of R2 also a letter. For the detailed rationale behind these requirements, see Moore (2020) and Moore et al. (2022). The requirements are:

R0: There is evidence of exposure to noise with a sufficient intensity and duration to have the potential for producing NIHL (Moore et al., 2022). This is usually the case for former military personnel when they have seen active service and have not always worn hearing protection, especially when the individual reports experiencing temporary hearing loss and/or tinnitus during service.

R1: A single value of the hearing threshold level (HTL) at 3, 4, 6, or 8 kHz is at least 10 dB higher than the HTL at 1 or 2 kHz. This is based on the observation that noise exposure during military service typically produces the greatest hearing losses at 4, 6, and 8 kHz, but sometimes produces the greatest loss at 3 kHz (Lowe & Moore, 2021; Moore, 2020).

R2a: The difference between HTLs at 8 and 6 kHz is at least 5 dB smaller than would be expected from age alone or the difference between HTLs at 8 and 4 kHz or between 8 and 3 kHz is at least 10 dB smaller than would be expected from age alone, based on the median values from ISO 7029 (2017). This resembles methods based on identifying a notch or bulge in the audiogram, but is based on the fact that noise exposure during military service typically leads to less hearing loss at 8 than at 6 kHz, and to similar hearing loss at 4 and 8 kHz (Lowe & Moore, 2021; Moore, 2020).

R2b: The HTL at any one of 4, 6, or 8 kHz is at least 20 dB higher than the median HTL for each frequency expected for that age, based on ISO 7029 (2017). The frequencies of 4, 6, and 8 kHz were chosen because these are the frequencies that are usually most affected by noise exposure during military service, but the exact frequency showing the greatest loss varies across individuals.

To reach a positive diagnosis of M-NIHL, R0 and R1 and either R2a or R2b (or both) must be satisfied.

Although the M-NIHL method was shown to have high sensitivity (Lowe & Moore, 2021; Moore, 2020), its specificity was only moderate (Moore & von Gablenz, 2021). This moderate specificity is probably a consequence of the high prevalence of mild hearing loss at high frequencies and the moderately high prevalence of small audiometric notches among the general population (Pudrith et al., 2022; Schlauch & Carney, 2011). However, it may also be partly a consequence of some problems with the M-NIHL method.

Consider first the R2a requirement of the M-NIHL method. The values of 5 and 10 dB were chosen based on the typical shapes of the audiograms of military personnel (Moore, 2020) so to give high sensitivity of the method, but they may not be optimal for achieving a good balance between sensitivity and specificity. Inspection of the results for individuals in the control population showed that false positives (diagnoses of NIHL when NIHL was not present) often occurred because the difference in HTLs at 6 and 8 kHz just met the minimum 5-dB criterion set in the M-NIHL method. In the present paper, the R2a requirement was modified in two ways. First, the criterion difference between HTLs at 8 and 6 kHz was required to be the same as the criterion difference between HTLs at 8 and 4 kHz or between HTLs at 8 and 3 kHz. Second, the magnitude of the criterion difference (i.e., the depth of the notch or bulge), instead of being fixed at 10 dB, was adjusted so as to give a good balance between sensitivity and specificity. The value of the depth of the notch or bulge is hereafter denoted Mag(Notch).

Consider next requirement R2b. There are two problems with this requirement. First, since it depends on the HTL at any one of three frequencies, it may be strongly affected by measurement errors at any one of those frequencies. Second, the margin of 20 dB at any one of three frequencies may be too small, since a hearing loss of 20 dB at a single high frequency (relative to ISO 7029) is common in the general population. To reduce these problems, R2b was modified to be based on the difference between the average measured HTL across 4, 6, and 8 kHz and the average expected HTL across 4, 6, and 8 kHz, based on ISO 7029. This difference is denoted hereafter Mag(ExcessHF). A criterion value of Mag(ExcessHF) giving a good balance between sensitivity and specificity was determined.

## Modifications to the M-NIHL Method

### Quantification of Mag(Notch) and Mag(ExcessHF)

The R2a requirement of the M-NIHL method was modified by creating a magnitude value corresponding to the size of the notch or bulge and then determining what magnitude value gave a good balance between sensitivity and specificity. The steps in calculating this magnitude value were as follows:

1. The median age-associated hearing loss (AAHL) values for the age of the individual concerned were calculated for frequencies from 3 to 8 kHz using the equations in ISO 7029 (2017). These are denoted AAHL(*x*), where *x* denotes frequency in kHz.
2. The following quantities were calculated:
  - [HTL(6) – HTL(8)] – [AAHL(6) + AAHL(8)]
  - [HTL(4) – HTL(8)] – [AAHL(4) + AAHL(8)]
  - [HTL(3) – HTL(8)] – [AAHL(3) + AAHL(8)]

where HTL(*x*) denotes the hearing threshold level of the individual at frequency *x*.

3. The maximum of (a), (b), and (c) was taken.

This quantity, Mag(Notch), is a measure of the size of the largest notch or bulge in the audiogram relative to the audiogram that would be expected from ISO 7029 (2017). Taking the maximum of (a), (b), and (c) allows for the fact that the notch, when present, is not always centered at 4 kHz but can also be centered at 3 or 6 kHz (Lowe & Moore, 2021; Moore, 2020).

R2b of the M-NIHL method was modified to be based on the average HTL across 4, 6, and 8 kHz. Specifically, the following was calculated to give Mag(ExcessHF):

$$\begin{array}{rl} \mathrm{Mag}(\mathrm{ExcessHF}) & =[\mathrm{HTL}(4)+\mathrm{HTL}(6)+\mathrm{HTL}(8)]/3\\ & \;\text{–}[\mathrm{AAHL}(4)+\mathrm{AAHL}(6)+\mathrm{AAHL}(8)]/3 \end{array}$$

The value of Mag(ExcessHF) indicates the extent to which the average high-frequency hearing loss is greater than the corresponding average AAHL values derived from ISO 7029 (2017). To help the reader who wishes to implement the rM-NIHL method, Table 1 gives the parameters for quadratic equations that can be used to calculate the AAHL values used to determine Mag(Notch) and Mag(ExcessHF).

**Table 1.** Parameters for Best-Fitting Quadratic, *y* = *a* + *bx* + *cx*^2^, for the ISO7029 AAHL Values Used in Computing Mag(Notch) and Mag(ExcessHF), Where *x* Denotes the Age of the Individual. All *r*^2^ Values for the Quadratics Were >0.998.

Sex	rM-NIHL requirement	Measure (*y*)	*a*	*b*	*c*
Male	Mag(Notch)	Sum HTL 6k8k	22.212	−1.895	0.041
	Mag(Notch)	Sum HTL 4k8k	20.080	−1.713	0.037
	Mag(Notch)	Sum HTL 3k8k	18.596	−1.590	0.034
	Mag(ExcessHF)	PTA468k	9.636	−0.837	0.018
Female	Mag(Notch)	Sum HTL 6k8k	20.507	−1.665	0.034
	Mag(Notch)	Sum HTL 4k8k	20.397	−1.617	0.032
	Mag(Notch)	Sum HTL 3k8k	18.957	−1.506	0.030
	Mag(ExcessHF)	PTA468k	9.907	−0.792	0.016

### Study Populations

Unfortunately, there is at present no “gold standard” for diagnosing M-NIHL with which a diagnostic method can be compared. The best that can be done is to evaluate sensitivity using a population that is highly likely to have M-NIHL and to evaluate specificity using a matched population that is unlikely to have had significant noise exposure. Here, as in Moore (2020) and Lowe and Moore (2021), audiograms of former military personnel were used to estimate sensitivity. All were men who had been on active duty in the British military and all were claiming compensation for M-NIHL, although for a few the primary complaint was tinnitus rather than hearing loss. All reported exposure to intense impulsive sounds, sometimes without hearing protection. Over 80% reported times when they had a temporary dulling of hearing and/or tinnitus following such exposure. None had a significant history of exposure to ototoxic substances or medications, or of current or previous ear diseases, significant head injury, or any relevant family history. None reported exposure to intense sounds other than during military service. Audiograms obtained before or close to the start of military service indicated no ear asymmetry ≥10 dB (based on the average HTL across 0.5, 1, 2, 3, 4, and 6 kHz), although the audiograms obtained after military service often showed marked asymmetry. Estimates of sensitivity were based on the assumption that the great majority of ears had M-NIHL, which may well not have been the case. Hence, sensitivity estimates represent lower bounds.

Specificity estimates were obtained using a control population (von Gablenz & Holube, 2016) carefully screened to exclude significant noise exposure, as described by Moore and von Gablenz (2021). The population was restricted to males aged between 29 and 60 years (the same as for the noise-exposed population) and their characteristics were matched as closely as possible to those of the noise-exposed population, except for the noise exposure. The control population had no excessive self-reported noise exposure, no self-reported history of ear diseases, no evidence of conductive hearing loss, and no ear asymmetry ≥10 dB (averaged over the frequencies 1, 2, 3, 4, and 6 kHz). Following screening based on these criteria, 189 individuals remained. As in Moore and von Gablenz (2021), one additional screening criterion was applied. All of the military noise-exposed men had HTLs ≤ 20 dB HL for all audiometric frequencies up to 6 kHz at the start of military service. Most of these men started military service when they were about 20 years old. Hence, a comparison sample without noise exposure should be screened to exclude those who were likely to have had HTLs >20 dB HL when they were aged 20 years. This was done based on the data of Linssen et al. (2014), as described in Moore and von Gablenz (2021). Only four men were excluded on this basis. Following screening, 185 individuals met the inclusion criteria. It was assumed that the great majority of this population did not have NIHL. However, since the individuals in the control population were not medically examined, some of them may have had causes of hearing loss other than noise exposure, so specificity estimates also represent lower bounds.

When developing a diagnostic method, it is desirable to use a database to evaluate the method that is different from the database used to develop it. Two sets of databases were used here. To estimate sensitivity, initially, a single database of military-noise-exposed individuals was created by merging the databases described in Moore (2020) and Lowe and Moore (2021) (138 individuals in total), with a new sample of 147 former military personnel, selected using the same criteria as in Moore (2020) and Lowe and Moore (2021), giving 285 individuals in total. The individuals in the new sample had, on average, less high-frequency hearing loss than those in the two earlier samples. Those in the new sample were largely based on individuals making claims for compensation over the past 2 years. The smaller hearing loss for this sample probably reflects an increasing trend for claims for compensation in the UK to be made by individuals with only mild hearing loss.

Next, to create two roughly equivalent databases of noise-exposed individuals, the order of individuals in the combined database was randomly shuffled, and then the database was split into two, with 143 in the first and 142 in the second. These are denoted MilDB1 and MilDB2. Similarly, the 185 individuals in the control database were randomly divided into two databases, one with 93 individuals and one with 92 individuals. These are denoted ContDB1 and ContDB2. The databases MilDB1 and ContDB1 were used to develop the rM-NIHL method. The databases MilDB2 and ContDB2 were used for evaluation of the method.

Means and standard deviations (SDs) of the HTLs of each group are given in Table 2. Note that on average both databases of military noise-exposed individuals had greater hearing loss at high frequencies for the left than for the right ears, as has been reported previously (Keim, 1969; Lowe & Moore, 2021; Moore, 2020). This has been attributed to greater noise exposure of the left ear, on average, for example, because of the way that a rifle is usually fired from the right shoulder, which usually results in partial shielding of the right ear via the head-shadow effect (Keim, 1969).

**Table 2.** Means (SDs) of the Ages and HTLs (dB HL) for Each Ear (R = Right, L = Left) of Each Database for Frequencies (in kHz) That Affect the rM-NIHL Diagnostic Method. The Number of Individuals in Each Group is Indicated by *n.*

Ear/Frequency (kHz)													
Group	Age	R 1	R 2	R 3	R 4	R 6	R 8	L 1	L 2	L 3	L 4	L 6	L 8
MilDB1 *n* = 143	45.4 (8.9)	16.9 (11)	22.8 (15)	32.7 (18)	41.0 (21)	44.7 (22)	42.2 (24)	19.2 (12)	25.8 (16)	41.8 (21)	50.4 (21)	53.2 (23)	51.0 (23)
MilDB2 *n* = 142	44.7 (9.6)	16.7 (11)	22.6 (15)	33.4 (19)	41.3 (20)	44.6 (22)	41.4 (24)	17.9 (11)	25.3 (17)	39.9 (20)	48.1 (21)	52.6 (23)	50.0 (24)
ContDB1 *n* = 93	41.8 (12)	4.4 (5.2)	5.6 (6.5)	7.7 (7.7)	9.7 (9.1)	13.4 (12)	20.2 (15)	3.1 (5.1)	5.1 (7.6)	9.4 (10)	12.7 (11)	14.7 (11)	19.9 (14)
ContDB2 *n* = 92	41.2 (11)	3.8 (4.6)	4.3 (5.4)	8.0 (7.7)	9.2 (10)	11.5 (11)	18.1 (13)	2.6 (5.2)	4.1 (6.0)	7.9 (7.7)	10.4 (11)	14.9 (12)	19.9 (16)

### Selection of Criterion Values of Mag(Notch) and Mag(ExcessHF)

In what follows, three cases were considered: (1) A positive diagnosis for either or both ears of a given individual required for a positive diagnosis for that individual; (2) A positive diagnosis for both ears of a given individual required for a positive diagnosis for that individual; (3) Diagnosis for each ear considered separately. To select criterion values of Mag(Notch) and Mag(ExcessHF), initially many pairs of values of Mag(Notch) or Mag(ExcessHF) were used to estimate sensitivity and specificity for these three cases, using MilDB1 and ContDB1. As expected, sensitivity decreased and specificity increased when the criterion values for Mag(Notch) or Mag(ExcessHF) were increased. For relatively large criterion values of Mag(Notch) and Mag(ExcessHF), for example, 18.5 and 23 dB, respectively, the specificity was high (0.989) but the sensitivity was relatively low (0.664), if a positive diagnosis was required for both ears. For relatively small criterion values of Mag(Notch) and Mag(ExcessHF), for example, 10.5 and 15.0 dB, respectively, the specificity was lower (0.860), but the sensitivity was higher (0.888), if a positive diagnosis was required for both ears.

**Figure 1.**
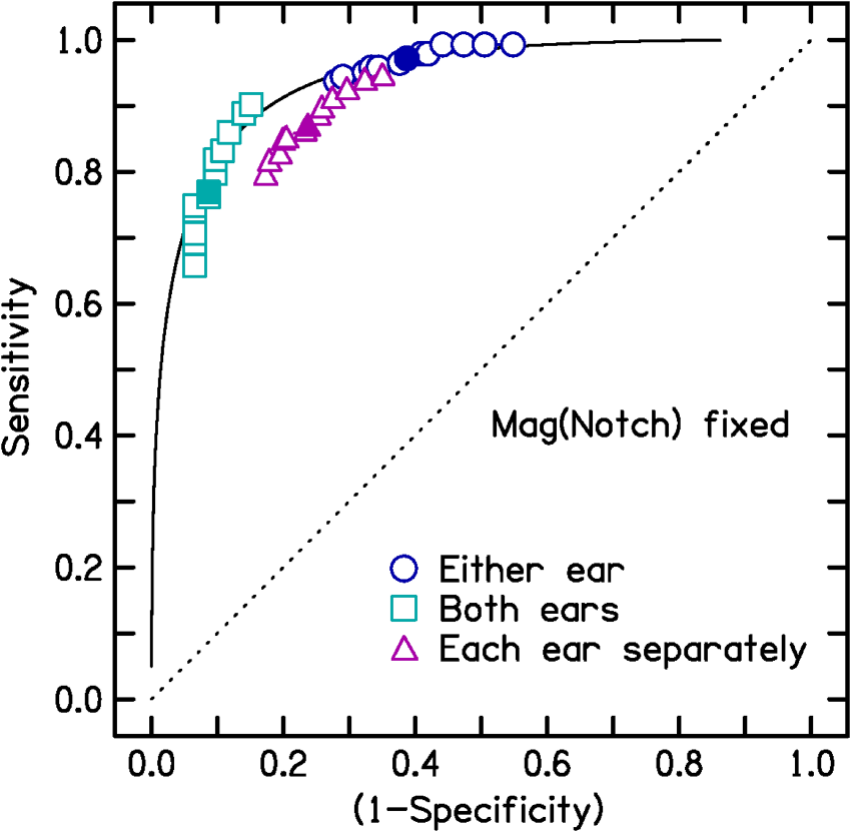
Receiver operating characteristic (ROC) curves generated keeping Mag(Notch) fixed at 14.5 dB and varying Mag(ExcessHF) from 13 to 25 dB, based on MilDB1 and ContDB1. Sensitivity is plotted against (1 − specificity). Three ROC curves are shown, corresponding to three cases: either or both ears (circles), each ear separately (triangles), and both ears (squares). In each case, the filled symbol is for the final chosen values of the variables [Mag(notch) = 14.5 dB, Mag(Excess HF) = 19 dB]. The ROC curves cover only a limited range because only plausible values of Mag(ExcessHF) were used. For comparison, the solid line shows an ROC curve for a fixed *d*′ value of 2.2, which was the value obtained for either or both ears with Mag(notch) = 14.5 dB and Mag(Excess HF) = 19 dB.

**Figure 2.**
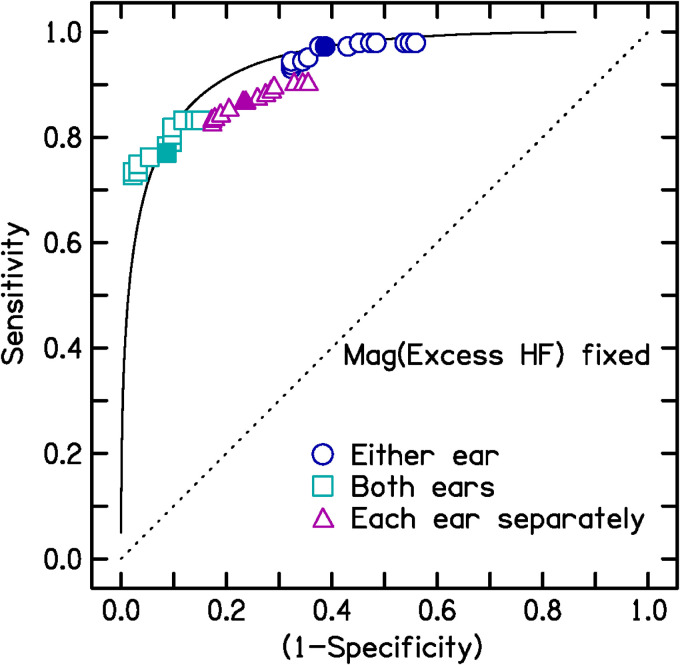
As Figure 1 but with Mag(ExcessHF) fixed at 19 dB and Mag(Notch) varied from 7.5 to 20.5 dB.

To examine the effects of varying Mag(Notch) and Mag(ExcessHF), two sets of Receiver Operating Characteristic (ROC) curves were generated. ROC curves are plots of sensitivity against (1-specificity), equivalent to plotting hits against false alarms in the terminology of signal detection theory (Green & Swets, 1974). In one set, Mag(Notch) was fixed at a value of 14.5 dB, and sensitivity and specificity were estimated as a function of Mag(ExcessHF) over the range 13 to 25 dB. In the other set, Mag(ExcessHF) was fixed at 19 dB and sensitivity and specificity were estimated as a function of Mag(Notch) over the range 7.5 to 20.5 dB. The resulting ROC curves are shown in Figures 1 and 2. Note that the sensitivity and specificity values do not cover the full range from 0 to 1, because only plausible ranges of the variable quantities Mag(ExcessHF) and Mag(Notch) were used.

A measure of the performance of a diagnostic method can be derived from the proportion of “hits” (sensitivity) and “false alarms” (1 − specificity):

$$d^{\prime }=Z(\mathrm{hit}\;\mathrm{rate})\text{–}Z(\mathrm{false}\;\mathrm{alarm}\;\mathrm{rate}),$$

where function *Z*(*p*), *p* ∈ [0,1], is the inverse of the cumulative Gaussian distribution (Green & Swets, 1974). The higher the value of *d'*, the better the performance of the method. Variations in the criterion magnitude of Mag(Notch) or Mag(ExcessHF) had only a small effect on *d'* values. For Mag(Notch) in the range 10.5 to 18.5 dB and Mag(ExcessHF) in the range 15 to 23 dB, *d'* values ranged from 2.05 to 2.61 based on a positive diagnosis for either ear; 2.06 to 2.52 based on a positive diagnosis for both ears; and 1.8 to 1.99 for each ear considered separately. Hence, the diagnostic performance of the rM-NIHL method was affected only slightly by the criterion values of Mag(Notch) and Mag(ExcessHF) when varied over a plausible range. To illustrate this, Figures 1 and 2 show ROC curves based on the assumption that *d*′ = 2.2, which was the value obtained for the case of a positive diagnosis for either ear (open circles) when Mag(Notch) was set to 14.5 dB and Mag(ExcessHF) was set to 19 dB. Note that the open circles in both figures fall close to the ROC curve for this fixed *d*′ value.

The final criterion values of Mag(Notch) and Mag(ExcessHF) were chosen to achieve as reasonable balance of probabilities. Specifically:

1. For ContDB1, for each ear considered separately, the proportion of false-positive values based on Mag(Notch) alone was similar to the proportion of false-positive values based on Mag(ExcessHF) alone.
2. Specificity based on a positive diagnosis for either ear of an individual was greater than 0.6.
3. Sensitivity based on a positive diagnosis for both ears was greater than 0.75.

The values that met these criteria were Mag(Notch) = 14.5 dB and Mag(ExcessHF) = 19.0 dB. With these values, for each ear considered separately the proportion of false-positive values based on Mag(Notch) alone was 0.124 and the proportion of false-positive values based on Mag(ExcessHF) alone was 0.108.

Table 3 shows values of sensitivity, specificity, and *d'* based on a positive diagnosis for either ear, both ears, and each ear considered separately. For comparison, Table 4 shows corresponding values for the M-NIHL method. As expected, sensitivity was slightly lower but specificity was markedly higher for the rM-NIHL method than for the M-NIHL method. Values of *d'* did not differ significantly for the two methods (Miller, 1996).

**Table 3.** Values of Sensitivity, Specificity, and *d'* for the rM-NIHL Method Using MilDB1 and ContDB1 Based on a Positive Diagnosis for (1) Either Ear; (2) Both Ears; (3) Each Ear Separately.

	Sensitivity	Specificity	*d'*
Either ear	0.972	0.613	2.20
Both ears	0.769	0.914	2.10
Each ear separately	0.871	0.763	1.85

**Table 4.** As Table 3 But for the Original M-NIHL Method.

	Sensitivity	Specificity	*d'*
Either ear	0.993	0.344	2.06
Both ears	0.930	0.828	2.42
Each ear separately	0.961	0.586	1.99

### Sensitivity and Specificity Values of rM-NIHL Using MilDB2 and ContDB2

Table 5 shows values of sensitivity, specificity, and *d'* for each of the three cases obtained using MilDB2 and ContDB2 with the selected criterion values of Mag(Notch) = 14.5 dB and Mag(ExcessHF) = 19 dB. The rM-NIHL method gave a reasonable balance between sensitivity and specificity. Table 6 shows values of sensitivity, specificity, and *d'* for the M-NIHL method using MilDB2 and ContDB2. As for the values obtained using MilDB1 and ContDB1, sensitivity was slightly lower but specificity was markedly higher for the rM-NIHL method than for the M-NIHL method. Again, values of *d'* did not differ significantly for the two methods (Miller, 1996). For the case that is most relevant in a medico-legal context in the opinion of the authors, namely a positive diagnosis for either or both ears, sensitivity for the rM-NIHL method (0.979) was only slightly lower than for the M-NIHL method (0.993), while specificity for the rM-NIHL method (0.630) was markedly higher than for the M-NIHL method (0.402). It can be concluded that, in a medico-legal context, the rM-NIHL method has a better balance between sensitivity and specificity than the M-NIHL method.

**Table 5.** Results for the rM-NIHL Method Using MilDB2 and ContDB2. Otherwise as Table 3.

	Sensitivity	Specificity	*d'*
Either ear	0.979	0.630	2.37
Both ears	0.761	0.946	2.32
Each ear separately	0.870	0.788	1.92

**Table 6.** As Table 5 but for the Original M-NIHL Method.

	Sensitivity	Specificity	*d'*
Either ear	0.993	0.402	2.21
Both ears	0.880	0.815	2.07
Each ear separately	0.940	0.609	1.83

## Discussion

The M-NIHL and rM-NIHL methods led to the same diagnosis in more than 90% of cases for the noise exposed former military personnel when each ear was considered separately. The exceptions occurred for cases where the diagnosis was very marginal. This is illustrated for an example ear in Tables 7 and 8. Requirement R1 was just met because the HTL at 6 kHz was 10 dB higher than at 1 and 2 kHz. For the M-NIHL method (Table 7), R2b was not met but R2a was just met, based on the measured HTLs at 6 and 8 kHz. Thus the diagnosis was positive. For the rM-NIHL method, the value of Mag(ExcessHF) was well below the criterion value and the value of Mag(Notch), 7.7 dB, was below the criterion value of 14.5 dB, so a positive diagnosis was not made. The rM-NIHL method can be regarded as more conservative for such marginal cases.

**Table 7.** Example of a Positive Diagnosis of NIHL Using the M-NIHL Method. Requirement R2b was not Met and Requirement R2a was Just Met on the Basis of the HTLs at 6 and 8 kHz.

Age, years	52						
Frequency, kHz		1	2	3	4	6	8
HTL, dB HL		10	10	10	10	20	15
AAHL, dB HL		4.6	7.5	10.1	12.4	15.9	18.6
Requirement R1 met?				FALSE	FALSE	TRUE	FALSE
RequirementR2a met?				FALSE	FALSE	TRUE	
Requirement R2b met?					FALSE	FALSE	FALSE

**Table 8.** Example of a Diagnosis Using the rM-NIHL Method for the Same Ear as Shown in Table 7. The Value of Mag(ExcessHF) Was Well Below the Criterion Value and the Value of Mag(Notch) Was Below the Criterion Value of 14.5 dB, so a Positive Diagnosis Was Not Made.

Age, years	52						
Frequency, kHz		1	2	3	4	6	8
HTL, dB HL		10	10	10	10	20	15
AAHL, dB HL		4.6	7.5	10.1	12.4	15.9	18.6
Magnitude R1, dB				0	0	10	5
Mag(Notch), dB				3.5	1.2	7.7	
Mag(ExcessHF), dB						−0.6	

This paper has emphasized the role of the audiometric configuration in the diagnosis of NIHL sustained during military service. However, other factors are also important. In particular, as mentioned earlier, it is important to obtain a full medical history to rule out possible causes of hearing loss other than noise exposure (Moore et al., 2022). It is also important to document the types and durations of noise exposures of the individual, to assess whether the individual experienced a dulling of hearing or tinnitus following the exposures (Brungart et al., 2019), and to assess whether other symptoms associated with noise exposure are present, such as tinnitus and hyperacusis (Lowe & Moore, 2021). A final diagnosis of NIHL for a particular individual should take account of these aspects as well as the audiometric configuration.

## Limitations

Some limitations of this study should be noted. First, the noise-exposed samples were not random or representative samples of former military personnel. The samples were restricted to those claiming compensation for noise-induced hearing damage. This increased the likelihood of them having M-NIHL, making them suitable for estimating the sensitivity of the rM-NIHL method, but is associated with the risk that the hearing loss was exaggerated. This risk is mitigated by the fact that all of the audiograms used in this paper were obtained according to the recommendations of the British Society of Audiology (2018), which meant that the procedure incorporated measures of response consistency. Another limitation is that the noise-exposed and control samples were not matched in terms of alcohol consumption, smoking, socio-economic status, or educational level, all of which are weakly associated with measured HTLs for a given age (Davis, 1995; Dawes et al., 2014). It should be noted, however, that these factors are very difficult to fully assess or quantify when diagnosing NIHL in a medico-legal context (Coles et al., 2000). Finally, the military noise-exposed groups were probably less highly screened than the control groups in terms of exposure to noise outside of military service. However, for the military noise-exposed groups, it seems likely that their exposure during military service far outweighed their exposure during other work or leisure activities (Jokel et al., 2019). This makes it likely that when M-NIHL was diagnosed, it was due primarily to noise exposure during military service rather than to noise exposure during other activities.

It should also be noted that the rM-NIHL method is intended to be applied to typical cases of military noise-exposed individuals who have greater hearing loss at high frequencies than at low frequencies. In rare cases, military personnel present with greater hearing loss at low than at high frequencies or with a “flat” or “near-flat” audiogram. This can be associated with exposure to intense low-frequency noise and vibration, as can occur inside a tank. In such cases, a failure to reach a positive diagnosis using the rM-NIHL method does not mean that NIHL is not present. Therefore, it is important also to take the type of noise exposure into account.

## Summary and Conclusions

The method of diagnosing NIHL sustained during military service proposed by Moore (2020) was modified so as to give a better balance between sensitivity and specificity. For the modified method, rM-NIHL, in addition to the *R*_0_ and *R*_1_ requirements of the original M-NIHL method, a positive diagnosis of NIHL is made for a given ear if either or both of the following requirements are met:

1. The maximum of
  - HTL(6) – HTL(8) – AAHL(6) + AAHL(8)
  - HTL(4) – HTL(8) – AAHL(4) + AAHL(8)
  - HTL(3) – HTL(8) – AAHL(3) + AAHL(8),

denoted Mag(Notch), should be ≥14.5 dB.

2. The value of
  $$\begin{array}{r} {}[\mathrm{HTL}(4)-\mathrm{AAHL}(4)+\mathrm{HTL}(6)-\mathrm{AAHL}(6)\\ +\;\mathrm{HTL}(8)-\mathrm{AAHL}(8)]/3, \end{array}$$

denoted Mag(ExcessHF), should be ≥19 dB.

If an individual is classified as having NIHL based on a positive diagnosis for either or both ears, based on MilDB2 and ContDB2 the rM-NIHL method has a sensitivity of 0.979 and a specificity of 0.630, giving a *d*′ value of 2.37.
